# RAC1b overexpression stimulates proliferation and NF-kB-mediated anti-apoptotic signaling in thyroid cancer cells

**DOI:** 10.1371/journal.pone.0172689

**Published:** 2017-02-24

**Authors:** Márcia Faria, Paulo Matos, Teresa Pereira, Rafael Cabrera, Bruno A. Cardoso, Maria João Bugalho, Ana Luísa Silva

**Affiliations:** 1 Unidade de Investigação de Patobiologia Molecular, Instituto Português de Oncologia de Lisboa Francisco Gentil E.P.E., Lisboa, Portugal; 2 BioISI–Biosystems and Integrative Sciences Institute, Faculdade de Ciências da Universidade de Lisboa, Portugal; 3 Instituto Nacional de Saúde, Doutor. Ricardo Jorge, Lisboa, Portugal; 4 Serviço de Anatomia Patológica, Instituto Português de Oncologia de Lisboa Francisco Gentil E.P.E., Lisboa, Portugal; 5 Serviço de Endocrinologia, Diabetes e Metabolismo, do CHLN—Hospital Santa Maria, Lisboa, Portugal; 6 ISAMB–Instituto de Saúde Ambiental, Faculdade de Medicina da Universidade de Lisboa, Lisboa, Portugal; Institute of Experimental Endocrinology and Oncology (IEOS), ITALY

## Abstract

Overexpression of tumor-associated RAC1b has been recently highlighted as one of the most promising targets for therapeutic intervention in colon, breast, lung and pancreatic cancer. RAC1b is a hyperactive variant of the small GTPase RAC1 and has been recently shown to be overexpressed in a subset of papillary thyroid carcinomas associated with unfavorable outcome. Using the K1 PTC derived cell line as an in vitro model, we observed that both RAC1 and RAC1b were able to induce a significant increase on NF-kB and cyclin D1 reporter activity. A clear p65 nuclear localization was found in cells transfected with RAC1b-WT, confirming NF-kB canonical pathway activation. Consistently, we observed a RAC1b-mediated decrease in IκBα (NF-kB inhibitor) protein levels. Moreover, we show that RAC1b overexpression stimulates G1/S progression and protects thyroid cells against induced apoptosis, the latter through a process involving the NF-kB pathway. Present data support previous findings suggesting an important role for RAC1b in the development of follicular cell-derived thyroid malignancies and point out NF-kB activation as one of the molecular mechanisms associated with the pro-tumorigenic advantage of RAC1b overexpression in thyroid carcinomas.

## Introduction

Thyroid cancer is the most common endocrine malignancy and its incidence has rapidly grown worldwide over the past few decades [1]. In Portugal, a significant annual increase of 2 to 9 per cent has been observed in its incidence from 1988 to 2011 [2].

The standard of care for thyroid carcinoma is surgery, regardless its histological subtype. Most thyroid malignancies are differentiated follicular cell-derived thyroid carcinomas (DTCs), comprising the papillary (PTC) and follicular (FTC) thyroid carcinoma subtypes [3,4]. These forms usually respond well to the conventional therapeutic protocol, which consists of thyroidectomy followed by TSH suppressive therapy and, eventually, radioactive iodine ablation therapy in selected patients accordingly to individual risk level [5]. There are, however, advanced forms of thyroid cancer that are surgically unresectable or unresponsive to radioiodine treatment. In those cases the therapeutic options are very limited and the 10*-*year survival rate drops from 60% to only 10% [6]. Thus, understanding the molecular mechanisms associated with tumor development and aggressiveness in order to identify novel targets for therapeutic intervention would be clinically relevant.

Activating alterations in the canonical Ras/Raf/MEK/ERK pathway (mitogen-activated protein kinase–MAPK pathway) are considered to have key role in thyroid carcinogenesis [4,7]. Although a single oncogenic alteration in MAPK pathway might be sufficient to drive thyroid cell tumorigenesis, additional molecular events are likely to be associated with thyroid malignancy progression leading to more aggressive phenotypes and poorer clinical outcomes. Our previous findings support that the overexpression of RAC1b, a highly activated variant of RAC1, may be one of these modifier events: we have shown that RAC1b is overexpressed in a subset of follicular cell-derived thyroid carcinomas compared to normal thyroid tissue and that this overexpression is significantly associated with a poorer outcome, in both PTC and FTC patients [8]. Nevertheless, although these findings support a role for RAC1b in thyroid tumorigenesis, the mechanisms by which RAC1b contributes to this malignancy has not been investigated.

RAC1b is a splice variant of the small GTPase RAC1, a member of the RAS superfamily of small GTP-binding proteins [9]. By switching between an inactive GDP-bound form and an active GTP-bound form [10–13] these factors regulate the activation of numerous signaling cascades that control several cellular processes, including cell proliferation and survival [11,13,14]. When compared to RAC1, RAC1b contains an additional domain in the vicinity of an important regulatory region of the GTPase [11]. This addition facilitates RAC1b activation and gives it selective downstream signaling. [15–17]. RAC1b has been described as potent activator of only some of the RAC1 downstream pathways, particularly classical NF-kB-mediated gene transcription [18,19]. Increased or constitutive NF-kB activation has been detected in several human malignancies, consistent with many of the NF-kB regulated genes being involved in cancer-associated processes. NF-kB activation has been also reported to play a role in thyroid malignancies, being associated with resistance to apoptosis and maintenance of the transformed phenotype [20–22]. The NF-kB family of transcription factors is composed of five members [RelA(p65), RelB, c-Rel, NF-kB1 (p50 and its precursor p100), NF-kB2 (p52 and its precursor p105)] and their activity is regulated by two major pathways referred as canonical and non-canonical. Contrary to RAC1 that activates both pathways, RAC1b selectively activates the canonical pathway [18], which regulates the activity of NF-kB dimers mostly composed of RelA(p65) and p50. These dimers are held in the cytoplasm in an inactive form bound to the specific inhibitor IkB (inhibitor of kB). In response to stimuli, the IKK (IkB Kinase) complex mediates the phosphorylation of IkB, inducing its degradation by the ubiquitin-proteasome pathway. This enables free, active NF-kB dimers to translocate to the nucleus and activate transcription of genes involved in several biological processes such as proliferation, migration and resistance to apoptosis [23]. RAC1b was previously shown to act as relevant player in the stimulation of cell cycle progression and cell survival through pathways involving NF-kB [18]. In thyroid cell systems, however, the impact of RAC1b signaling towards the NF-kB pathway has not been investigated. With the aim of getting insights into the molecular mechanisms associated with RAC1b overexpression and downstream signaling in thyroid tumorigenesis, we studied the effect of RAC1b in the activity of NF-kB as well as its impact in cellular processes associated with cancer development and progression such as cell cycle progression and resistance to apoptotic stimuli.

## Materials and methods

### Cell lines, plasmid constructs and transfections

The human PTC-derived cell line K1 was maintained in Dulbecco's Modified Eagle Medium/Nutrient Mixture F-12 (DMEM: F12, 1:1,Gibco, Gaithersburg, MD, USA) supplemented with 10% (v/v) fetal bovine serum (FBS) (Biochrom, Cambourne, UK), 1% (v/v) GlutaMax (Gibco), 1.25% (v/v) sodium bicarbonate (Gibco) and 0.5% (v/v) sodium pyruvate (Gibco).The cell line Nthy-ori 3–1 (Nthy), derived from human thyroid follicular normal epithelium, was maintained in RPMI-1640 HEPES modified medium (Sigma, St.Louis, MO, USA), supplemented with 10% (v/v) FBS and 1% (v/v) GlutaMax.

The plasmids used in this study [19,24] were a kind gift from Dr. Peter Jordan, from Instituto Nacional de Saúde Dr. Ricardo Jorge: pcDNA3-HA-IκBα(A32A36), -964-CycD1-Luc vector, 3x-kB-luc vector, pRL-TK reporter (the internal control reporter constitutively expressing Renilla luciferase), pEGFP-RAC1b-L61, pEGFP-RAC1-L61, pEGFP-RAC1b-WT, pEGFP-RAC1-WT. The pEGFP-C1 (BD Biosciences Clontech, Mountain View, CA, USA) and the pcDNA3.1 (+)-empty vector (Invitrogen, Waltham, MA, USA) were used to generate mock controls.

Transfections were carried out using Lipofectamine 2000 (Invitrogen), according to the manufacturer’s instructions. Cells seeded in 35mm dishes at 70–85% confluence were transfected with 2μg of total plasmid DNA. The amounts used of RAC1/1b constructs were optimized to achieve similar levels of protein expression (when required, the total amount of DNA was adjusted with empty vector). For the NF-kB and cyclin D1 reporter activity assays, the RAC1/1b constructs were co-transfected with 0.1μg of the pRL-TK (Renilla luciferase) reporter and 1μg of NF-kB or cyclin D1 reporters. In experiments using the mutant non-degradable “super-repressor” IκB protein (IκBaa), the RAC1/1b constructs were co-transfected with 100ng of pcDNA3-HA-IκBα (A32A36).

### NF-kB and cyclin D1 reporter activity assays

For NF-kB and cyclin D1 reporter activity experiments cells were harvested 24h after transfection and lysed with passive lysis buffer 1x (Promega, Madison, WI, USA) following the manufacturer’s instructions. When applicable, cells were incubated with the RAC family small GTPases inhibitor EHT 1864 (final concentration 0.1mM) for 4h before harvesting. Luciferase activity reporter assays were performed using the Dual-Luciferase® Reporter (DLR™) Assay System (Promega) following the manufacturers' instructions. Lysates were assayed in duplicate and additional aliquots were analyzed by Western blot to assess RAC1/1b protein expression levels.

### Total protein lysates, nuclear/cytosolic extracts and western blot

Protein extracts by total cell lysis were obtained using RIPA (Radio-Immunoprecipitation Assay) buffer. Nuclear and cytosolic extracts were prepared as described [25].Briefly, cells were lysed in cytosol fractionating buffer [50mM HEPES (pH 7.2), 2mM EDTA, 10mM NaCl, 250mM sucrose, 2mM DTT, 0.1% (v/v) Nonidet-P40 and a protease inhibitor cocktail (Sigma)] and the cytosolic protein extract was obtained by collecting the supernatant by centrifugation. The nuclear protein extract was obtained by lysing the pellet in nuclei fractionating buffer [50mM HEPES (pH 7.2), 2mM EDTA, 400mM NaCl, 20% (v/v) glycerol, 2mM DTT and protease inhibitor cocktail]. Protein samples were prepared for western blot, resolved in 12% SDS–PAGE and transferred to PVDF membranes (Bio-Rad, Hercules, CA, USA), according to standard protocols. Primary antibodies used in this study were mouse monoclonal anti-RAC1 (Millipore; Temecula, CA, USA) at1:2000 dilution, rabbit polyclonal anti-NF-kB p65 NLS (Thermo Scientific, Waltham, MA, USA) at1:1000 dilution, rabbit polyclonal anti-IκBα (Santa Cruz Biotechnology, Dallas, TX) at1:1000 dilution, mouse monoclonal anti-β-actin (Sigma) at 1:10000 dilution and rabbit polyclonal anti-Histone H2B (Millipore) at 1:1000 dilution. Detection was carried out using secondary peroxidase-conjugated anti-mouse IgG (Bio-Rad) or anti-rabbit IgG (Bio-Rad) antibodies followed by chemiluminescence.

### Confocal immunofluorescence microscopy and immunohistochemistry

For imunofluorescence analysis K1 cells were grown on 10 X 10mm glass cover slips and transfected as described above. Immunofluorescence was performed as previously described [19]. NF-kB-p65detection was carried out using rabbit anti-NF-kB p65 NLS antibody (Thermo Scientific) at 1:750 dilution (4°C, overnight) followed by goat anti-rabbit secondary antibody, Alexa Fluor® 532 (Life Technologies, Waltham, MA, USA) at 1:500 dilution. Images were recorded with the 405-nm, 488-nm, and 532-nm laser lines of a Leica TCS-SPE confocal microscope and processed with Adobe Photoshop software. Immunohistochemical detection of NF-kB-p65 protein was performed on archived formalin-fixed, paraffin embedded PTC samples. The selected PTCs have been previously assessed for RAC1b expression and BRAF mutational status [8]. NF-kB detection was performed in 4 RAC1b-positive and 4 RAC1b-negative tumors (1 out of 4 RAC1b-positive and 1 out of 4 RAC1b-negative tumors carried BRAF V600E mutation); the adjacent normal tissue in each tumor sample was analyzed as control. Immunostaining was performed manually using the peroxidase-indirect-polymer method (Dako, Code 5007 Glostrup, Denmark). 3μm thick sections were cut from paraffin-embedded routine tissue blocks, and transferred into Superfrost plus slides. Sections were de-waxed, rehydrated and subjected to epitope antigen retrieval (20 minutes, 94°C) with Dako Envision Flex Target Retrieval Solution High pH 50x (Code K8004) in a pre-treatment module PTlink (Dako, Model PT 10130). Primary polyclonal anti-NF-kB p65 NLS antibody (Thermo Scientific, 1:3000, overnight) was used. Endogenous peroxidase was inactivated with peroxidase blocking solution (Dako, code S2023) for 15 minutes. Counterstaining was performed with Mayer’s haematoxylin. For negative controls, primary antibodies were omitted during the staining.

### Cell cycle and cell death analysis by flow cytometry

K1 cells were transfected as described above. For cell cycle experiments, 6 hours after transfection, cells were synchronized by serum starvation (1%FBS, DMEM: F12 for 12h) and then stimulated (10% FBS, DMEM: F12 for 6 hr), fixed and permeabilized. The fraction of cells in each phase of cell cycle (G1, S, and G2/M) was determined by DNA content analysis using propidium iodide staining (PI—50μg/mL) followed by flow cytometry. For cell death analysis, 24 hours after transfection cells were incubated in fresh medium for two additional hours in the absence or presence of staurosporine (Santa Cruz Biotechnology) at selected final concentrations (5nM and 50nM). Stages of cell death were determined by flow cytometry, based on the combined cell staining with PI (2μg/mL) (Sigma, USA) and Annexin V (0.75μg/mL) (BioLegend, San Diego, CA; USA). Acquisition parameters were defined with the software BD Cell Quest; cells were acquired in the flow cytometer (FACScalibur–Becton Dickinson); acquired data was analysed using FlowJo (Tree Star Inc, USA) software.

### Statistical analysis

Statistical analysis was carried out using GraphPad Prism 5 software (San Diego, USA). Whenever appropriate, values are expressed as mean ± SD (Standard deviation). The two-tailed Student’s t-test or two-tailed Fisher’s exact test and the paired two-tailed Student’s t-test were used to perform statistical analysis and evaluate statistical significance of results (accepted as p<0.05).

## Results

### RAC1b stimulates canonical NF-kB reporter activation

**Fig 1:**
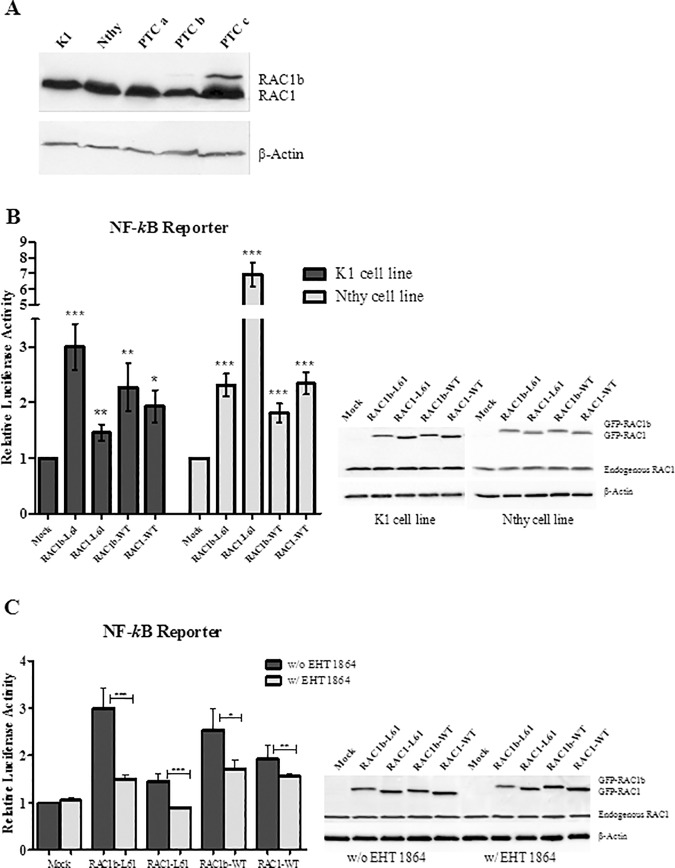
Luciferase activity of NF-kB regulated reporter in transfected K1 and Nthy cells. (A) Western blot documenting endogenous RAC1/1b expression in K1 and Nthy cell lines; RAC1b- overexpressing and non-overexpressing PTC samples were included as controls (PTC a, PTC b and PTC c). (B) Luciferase reporter activity driven by the NF-κB consensus motif upon expression of transfected GFP-RAC1/1b in K1 and Nthy cells. Western Blot documenting expression levels of transfected GFP-RAC1/1b in both cell lines. Beta-actin was used as a loading control. (B) Activity of NF-kB regulated reporter in K1 cells transfected with RAC1/1b variants upon treatment with EHT 1864. Western Blot documenting the expression of transfected RAC1/1b variants. RAC1/1b expression was assessed using anti-RAC1 primary antibody which detects both RAC1 and RAC1b variants Data are mean ± error bars (SD) of at least three independent experiments. Comparison of RAC1/1b effects with mock control was performed using a two-tailed Student’s t-test. A paired two-tailed Student’s t-test was used to evaluate the effect of EHT 1864 comparing treated with non-treated cells; p-values: *p≤0.05 **p ≤0.01 ***p ≤0.001.

Due to its high activity and selective signaling properties, RAC1b was shown to be a potent inducer of the canonical NF-kB pathway in colorectal cancer cells [18]. Here, we addressed the impact of RAC1b overexpression on NF-kB pathway, in the context of thyroid malignancies. For that we selected the normal (Nthy) and PTC-derived (K1) cell lines, which do not express detectable levels of RAC1b (Fig 1A), and tested the activity of a luciferase reporter regulated by the NF-kB-consensus motif in the presence and absence of RAC1b ectopic expression (Fig 1B). Cells were transfected with the luciferase-based reporter vector and either with the RAC1 or RAC1b wild-type forms or the constitutively active mutants, RAC1- and RAC1b-L61. The overexpression of RAC1b-WT and RAC1b-L61 in both PTC and normal cell lines induced a significant increase on reporter activation relatively to mock-transfected, control cells. Notably, in Nthy cells, RAC1 induced a more effective reporter response than RAC1b. Conversely in PTC-derived K1 cells RAC1b induced a considerably higher increase in reporter activity than that produced by RAC1 (Fig 1B). In addition, treatment with the RAC1/RAC1B selective inhibitor EHT1864, abrogated the observed effects in NF-kB reporter activity (Fig 1C), supporting the role of RAC1b in the stimulation of the NF-kB pathway in both cell lines.

### RAC1b downregulates IkBα and stimulates p65 (RelA) translocation to the nucleus

**Fig 2:**
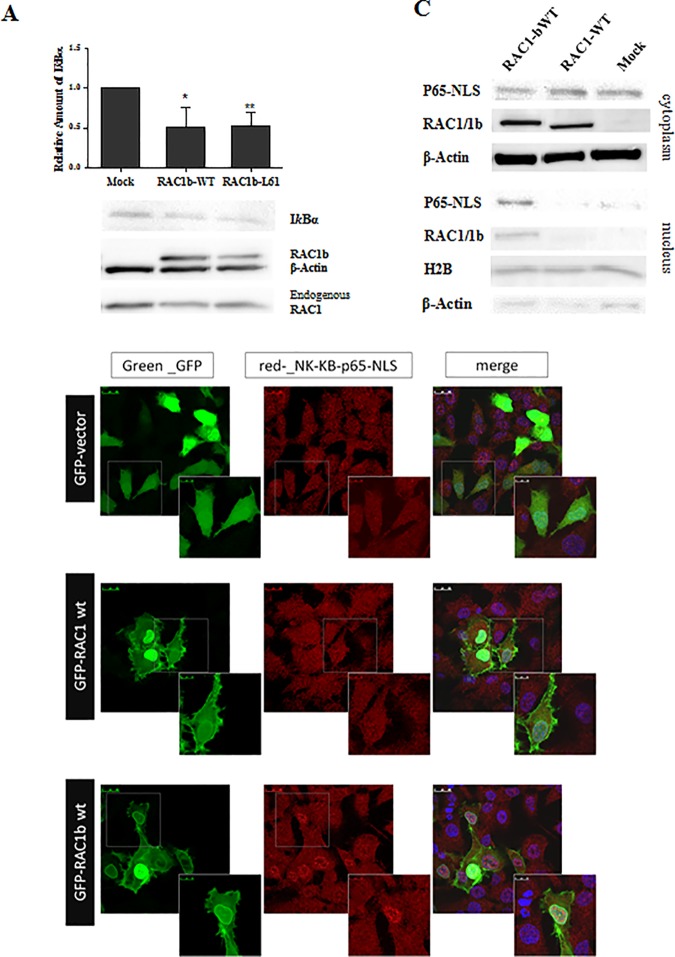
Effect of RAC1b overexpression on the subcellular location of NF-kB-p65 subunit. K1 cells were transfected with pEGFP-RAC1b-WT, pEGFP-RAC1-WT, or pEGFP-empty vector (Mock). (A) Western blot documenting IκBα levels upon GFP-RAC1b transfection in K1 cells. Protein levels were determined by densitometry using ImageJ software on blots from three independent experiments and results are expressed as mean values; two-tailed Student’s t-test was used to compare the effect of RAC1b expression with Mock control; p-values: *p≤0.05 **p ≤0.01 ***p ≤0.001. (B) Immunofluorescence confocal microscopy using anti-NF-kB p65-NLS antibody; nuclei were counterstained with DAPI. The overlay image of the DAPI, GFP and Red channels is shown. (C) Western Blot accessing NF-kB-p65-NLS in nuclear and cytoplasmic cell fractions. Histone H2B and β-Actin were used as nuclear and cytoplasmic loading controls, respectively.

To further confirm the RAC1b impact on the activation of the canonical NF-kB pathway in K1 cells, we study the influence of RAC1b on total IκBα protein expression (a negative regulator in the canonical NF-kB pathway) that is inversely correlated with the activation of NF-kB transcription factor. We observed a decrease of total IκBα levels upon ectopic overexpression of RAC1b (either WT or Q61L) (Fig 2A). The impact of RAC1b overexpression on the activation of the transcription factor NF-kB was further addressed by accessing its effect on the nuclear translocation of p65. Firstly, the effect of RAC1b on the subcellular localization of p65 was accessed by immunofluorescence using an antibody that binds the uncovered NLS region of p65 (i.e. the active p65 dimers). K1 cells were transfected with GFP-RAC1-WT, GFP-RAC1b-WT or control GFP-empty vector. As shown in Fig 2B, cells transfected with RAC1b-WT revealed a clear nuclear localization of p65, compared to non-transfected or to GFP transfected cells. In contrast, RAC1-WT had little effect on the accumulation of p65 in the nucleus. This observation was further confirmed by subcellular fractionation experiments where an increase on chromatin-associated p65 was clearly detected in the nuclear fraction upon the expression of RAC1b, but not of RAC1 (Fig 2C). All these observations are consistent with the effects observed in the activity of the NF-kB reporter.

**Fig 3:**
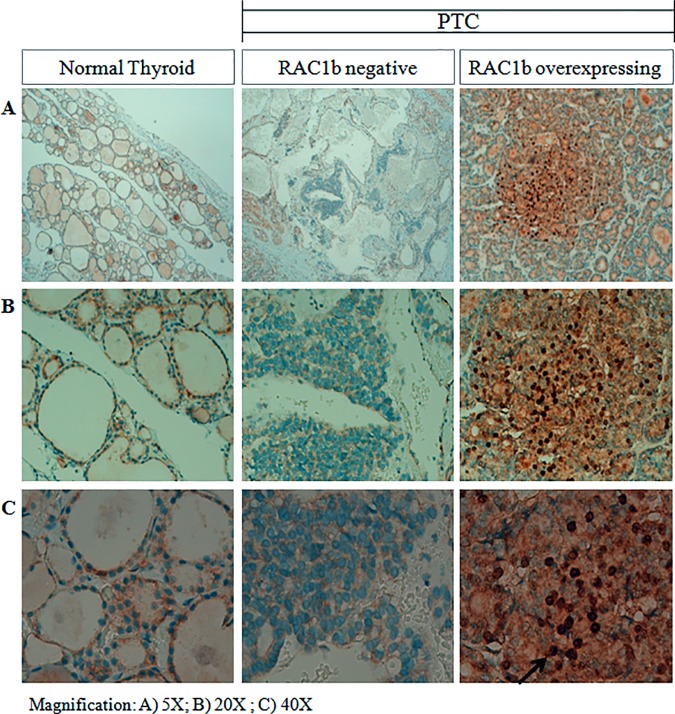
Immunohistochemical analysis of NF-kB in RAC1b-positive and RAC1b-negative PTC samples. NF-kB p65-NLS immunostaining patterns in representative normal thyroid and in RAC1b-overexpressing or RAC1b-negative tumor samples A positive nuclear staining was found to be prevalent in RAC1b-overexpressing tumors. Images were acquired with a Leica DMD 108 microscope; magnification: A) 5X; B) 20X; C) 40X.

To assess the biological significance of these findings we investigated whether p65 nuclear localization could distinguish RAC1b-positive from RAC1b-negative PTC tumor samples. Using immunohistochemistry we tested paraffin-embedded tissue specimens representative of both tumor types and evaluate differences in subcellular localization of the p65-NLS protein. As illustrated in Fig 3, the nuclear localization of NF-kB/p65 was found to be prevalent in RAC1b-overexpressing tumors when compared to those that are negative for RAC1b expression.

### RAC1b protects PTC-derived cells against apoptosis via the NF-kB pathway

Since one of the most relevant cancer-related effects of NF-kB stimulation is anti-apoptotic response, the impact of RAC1b overexpression in this process was further addressed.

**Fig 4:**
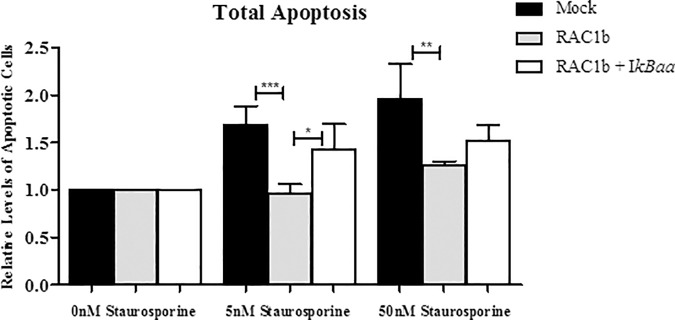
Effect of RAC1b overexpression on apoptosis. (A) Apoptotic cell death assessed by flow cytometry in K1 cells transfected with RAC1/1b variants upon apoptosis induction with increasing amounts of the proapoptotic agent, staurosporine. Levels of apoptosis were also evaluated upon co-expression of the non-degradable super-repressor IκBαA32A36 (IκBaa). Data are means ± error bars (SD) of at least three individual experiments. Comparisons were made using a two-tailed Student’s t-test. *p≤0.05 **p ≤0.01 ***p ≤0.001.

K1 cells were subjected to different concentrations of staurosporine and the proportion of apoptotic cells evaluated by flow cytometry in the presence and absence of RAC1b expression (Fig 4). As expected, the proportion of apoptotic mock-transfected cells increased with increasing concentrations of staurosporine. In contrast, RAC1b- expressing cells were clearly resistant to apoptotic stimuli, even when treated with the highest concentration of staurosporine. This resistance was abated by co-expression of the non-degradable IκBαA32A36 super-repressor mutant (IkBAA), indicating that the observed RAC1b-induced apoptosis resistance operates through the canonical pathway of NF-kb activation.

### RAC1b stimulates cyclin D1 reporter and cell cycle progression by a mechanism that is independent of NF-kB

**Fig 5:**
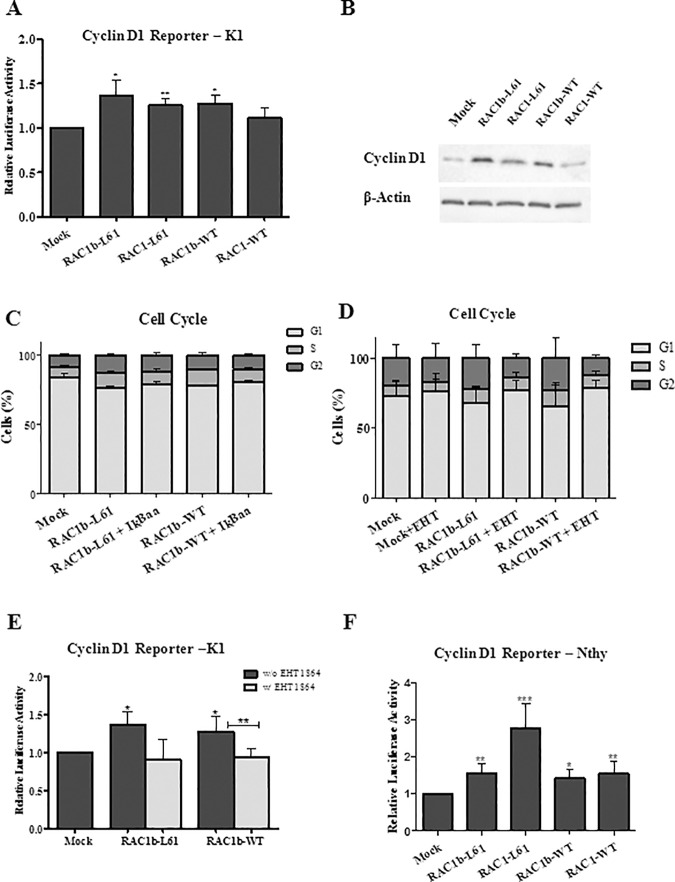
Effect of RAC1b overexpression on Cyclin D1 reporter and cell cycle progression. (A) Effect of RAC1/1b variants on luciferase activity derived from cyclin D1 regulated reporter in K1 cells. (B) Western blot documenting the protein levels of Cyclin D1 upon expression of RAC1/1b variants. (C) Flow cytofluorometric analysis of cell cycle assessed in the presence/absence of IκBaa. (D) Flow cytofluorometric analysis of cell cycle in the presence/absence of EHT 1864. The percentages of cells in each phase of cell cycle is represented. (E) Effect of RAC1b on luciferase activity derived from cyclin D1 regulated reporter in the presence/absence of the specific RAC inhibitor EHT 1864. (F) Effect of RAC1/1b variants on luciferase activity derived from cyclin D1 regulated reporter in Nthy cells. Data are means ± error bars (SD) of at least three individual experiments. Comparisons with the Mock control in each condition were performed using a two-tailed Student’s t-test; p-values: *p≤0.05 **p ≤0.01 ***p ≤0.001.

Since a role for RAC1b signaling in cell cycle progression that involves NF-kB activation and cyclin D1 expression has been previously reported [18], we tested whether this effect was relevant in a PTC biological system. We started to test the ability of RAC1/1b to stimulate the activity of a Cyclin D1 reporter in K1 cells. Both the WT and L61 forms of either RAC1 or RAC1b increased luciferase activity over control cells (Fig 5A). Also, an increase on protein levels of Cyclin D1 was observed upon expression of RAC1b variants compared to mock control (Fig 5B). The Cyclin D1 reporter induced activity, however, was similar for the different RAC1/1b forms and modest in magnitude (1.3-fold) as compared to that obtained for the NF-kB reporter (2 to 3-fold). This led us to address whether the effect of RAC1b on the cyclin D1 reporter was sufficient to impact cell cycle progression. Notably, we observed an increase in cell populations in S/G2 upon ectopic expression of either RAC1b-wt or–L61 (Fig 5C and 5D). However, while these effects were clearly reverted by co-treatment with the RAC1b inhibitor EHT 1864 (Fig 5D and 5E) neither was affected by co-expression of the IkBAA super-repressor (Fig 5C). Altogether, these results indicate that the mechanism of cell cycle progression induced by RAC1b works independently of NF-kB activation. Lastly, we aimed to ascertain whether the ability of RAC1b to induce cyclin D1 is still present in a different thyroid cell system, acting independently of the constitutive activation of BRAF V600E/MAPK pathway. To address this point, the effect of RAC1b in cyclin D1 reporter was additionally accessed in Nthy cells, which do not harbor BRAF V600E or other MAPK activating mutations. In this cell system, RAC1b was still able to induce cyclin D1 reporter activity to levels higher than that of mock control (Fig 5F), indicating that RAC1b-induced cyclin D1-associated cell cycle effects may work on thyroid cell systems even in the absence of the BRAF V600E/MAPK- activational effects.

## Discussion

The standard of care for differentiated follicular cell- derived thyroid carcinomas includes thyroidectomy, TSH suppressive therapy and eventually radioactive iodine treatment for selected patients accordingly to individual risk. Despite being usually associated with a good prognosis the rate of recurrence is high and a subset of patients presents radioiodine refractory and non-responsive neoplasias. So, the search for new therapeutic alternatives, as well as new diagnostic/prognostic markers is clinically relevant.

We have previously shown that RAC1b is overexpressed in a subset of thyroid follicular cell-derived carcinomas (of either PTC or FTC subtypes) associated with poorer outcomes. These findings suggest a role for RAC1b in the modulation of thyroid malignant progression. However, the mechanism by which RAC1b signaling impacts on thyroid tumorigenesis remained elusive. Similarly, several lines of evidence have implicated NF-kB in thyroid cancer progression but the mechanism leading to NF-KB activation in thyroid tumorigenesis is still poorly defined [20,26]. Here, we hypothesized that RAC1b might contribute for this process since RAC1b has been also shown to be a potent activator of the canonical NF-κB regulatory pathway in colorectal cancer. Therefore, in this study we addressed whether RAC1b retains the ability to stimulate NF-kB activation in thyroid biological systems and if this activation would be relevant for thyroid tumorigenesis.

Using the human PTC- derived K1 cell line as in vitro model, we observed that both RAC1 and RAC1b were able to induce a significant increase on NF-kB reporter activity. Also, a clear p65 nuclear localization was found in cells transfected with RAC1b-WT, confirming the activation of the NF-kB canonical pathway. Importantly, we confirmed that these in vitro observations were present in patient-derived samples, since nuclear-localized NF-kB was prevalent in RAC1b-overexpressing PTCs when compared to RAC1b- negative tumors. These findings prompted us to further assess the cellular consequences of RAC1b-mediated NF-kB activation in a thyroid biological system. Consistent with previous observations in other cell types, we observed that RAC1b expression significantly increased the resistance of PTC cells to apoptotic stimuli via stimulation of the NF-kB pathway [18,19]. Surprisingly, however, RAC1b ability to stimulate Cyclin D1and G1/S progression in PTC is independent of NF-kB activation, which is in contrast to previous observations in other carcinomas, namely colorectal carcinomas [18]. This suggests that in the thyroid biological context other RAC1b-stimulated pathways contribute to cell proliferation. Notably, we found additional indications of different RAC1b signaling contexts between colorectal and thyroid malignancies. Here we showed that in the PTC cells RAC1b induces NF-kB activation to substantially greater levels than RAC1, whereas in colorectal cancer cells RAC1 appears to be a more effective NF-kB activator than RAC1b [19]. Moreover, our results in thyroid indicate that the biological context of RAC1b signaling may also differ between normal and cancer tissues. In fact, in cells derived from normal follicular epithelium, RAC1 prevails over RAC1b in what concerns their signaling to the NF-kB pathway. This suggests that RAC1b overexpression acquires pro-tumorigenic relevance only in the context of an already altered cellular background. When we tested two other available PTC-derived cell lines, BCPAP and TPC1 (neither of which express RAC1b; S1A Fig), we found the NF-kB pathway to be so highly stimulated that even in basal conditions (mock control transfections) the luciferase reporter levels exceeded those of K1 cells overexpressing RAC1b-L61 (S1B Fig). Moreover, no significant reporter variations were observed in these cell lines upon overexpression of either RAC1 or RAC1b constitutively active mutants. This is consistent with the cellular background influencing NF-kB activity and with the hypothesis that the right context is required for RAC1b overexpression to become relevant in thyroid tumorigenesis. Although both K1 and BCPAP cells harbor the BRAF V600E mutation they present different NF-kB activation profiles. This suggests that BRAF activating mutations alone do not rule NF-kB activational status. K1 also harbors a PIK3CA E545K mutation which could be generating the right background to promote signaling through RAC1b, since this variant is much more easily activated that RAC1 [16]. However, TPC1 cells harbor a RET/PTC1 rearrangement which signals both trough BRAF and PI3K, and presented a NF-kB activation profile much more similar to BCPAP than to K1. This indicates that despite the reported synergy between RAC1b and BRAF V660E in colorectal tumors [27], the altered molecular background that promotes a RAC1b-induced NF-kB-mediated pro-oncogenic response in thyroid cancer remains elusive and probably goes beyond bone-fide genetic alterations in genes such as BRAF, PI3K or RET. Nonetheless our data indicate that during the malignant progression of some thyroid tumors the arising of RAC1b overexpression may suffice to offer tumor cells an additional survival advantage through NF-kB stimulation. This, in turn, can lead to more aggressive phenotypes and poorer clinical outcomes, as reported in both PTCs and FTCs [8,28].

In conclusion, taken together, our findings suggest an important role of RAC1b overexpression signaling through NF-kB in PTC tumorigenesis through apoptosis resistance. Moreover, our results indicate that RAC1b may also impact PTC cell proliferation through yet uncharacterized pathways. Together with the identified differences in RAC1b signaling between normal and neoplastic thyroid cells, our data open new research avenues for the study of small GTPase signaling in the context of thyroid cancer.

## Supporting information

S1 Fig Comparison of NF-κB regulated reporter activity in N-Thy, K1, BCPAP and TPC1 thyroid cell lines. (A) Western blot documenting endogenous RAC1b expression in K1, Nthy, BCPAC and TPC1 cell lines; a RAC1b- overexpressing PTC sample was included as control (PTC RAC1b+). (B) Luciferase reporter activity driven by the NF-κB consensus motif in basal (mock control transfection) conditions and upon expression of either GFP-RAC1b-L61 or GFP-RAC1-L61 in K1, Nthy, BCPAP and TPC1 cell lines. Data are mean ± error bars (SD) of at least three independent experiments.
(TIF)
