# Supplementary figures and images for: RAC1b overexpression stimulates proliferation and NF-kB-mediated anti-apoptotic signaling in thyroid cancer cells
Source: PLoS One. 2017 Feb 24;12(2):e0172689. doi: 10.1371/journal.pone.0172689 (PMC5325471; doi:10.1371/journal.pone.0172689)

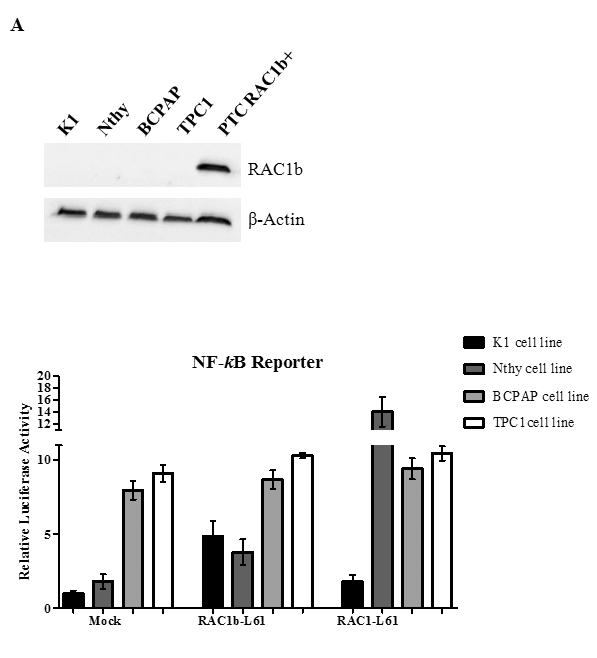

Supplement: S1 Fig — (A) Western blot documenting endogenous RAC1b expression in K1, Nthy, BCPAC and TPC1 cell lines; a RAC1b- overexpressing PTC sample was included as control (PTC RAC1b+). (B) Luciferase reporter activity driven by the NF-κB consensus motif in basal (mock control transfection) conditions and upon expression of either GFP-RAC1b-L61 or GFP-RAC1-L61 in K1, Nthy, BCPAP and TPC1 cell lines. Data are mean ± error bars (SD) of at least three independent experiments. (TIF) [file pone.0172689.s001.tif]
